# Objective detection of visual field defects with multifrequency VEPs

**DOI:** 10.1007/s10633-023-09949-4

**Published:** 2023-09-26

**Authors:** Katja Crux, Cord Huchzermeyer, Jan Kremers, Folkert K. Horn

**Affiliations:** 1 Department of Ophthalmology, University Hospital Erlangen, Schwabachanlage 6, 91054 Erlangen, Germany; 2 Department of Ophthalmology, University Eye Hospital, Friedrich-Alexander University Erlangen-Nürnberg, Schwabachanlage 6, 91054 Erlangen, Germany

**Keywords:** Steady state, Quadrant multifrequency VEP, Pattern reversal, Objective visual field test, Signal-to-noise ratio

## Abstract

**Purpose:**

To correlate multifrequency pattern reversal VEPs in quadrants (QmfrVEPs) with perimetric field losses for objective detection of visual field losses.

**Methods:**

QmfrVEP measurements were performed using four LED-based checkerboard stimulators to stimulate the four quadrants of the visual field. QmfrVEPs were measured monocularly in 5 normal subjects and in 5 glaucoma patients who showed losses in conventional Octopus perimetry. The pattern reversal frequency varied slightly between the stimulators: (11.92, 12.00, 12.08 and 12.16 reversals/sec). The responses to the different stimuli were identified by discrete Fourier analysis. VEPs were recorded using different electrode configurations, and the recording with the highest signal-to-noise ratio (SNR) was used for further analysis.

**Results:**

QmfrVEP responses from the different quadrants can be reliably measured and separated using the 0.08 reversals/sec interstimulus reversal frequency differences. The signal-to-noise ratio in the four quadrants was significantly correlated with the equivalent visual field losses obtained with perimetry (Spearman rank correlation: *P* < 0.001). In the five glaucoma patients, the SNR was reduced in 15 out of the 16 quadrants with a perimetric defect, in comparison to the results in quadrants of healthy subjects. This confirms the sensitivity of the procedure.

**Conclusion:**

QmfrVEP responses can be measured reliably. This pilot study suggests that high SNR values exclude visual field defects and that focal defects can be identified in glaucoma patients.

*Trial registration*: www.clinicaltrials.gov. NCT00494923.

## Introduction

Conventional perimetry is a psychophysical procedure that requires the patient's cooperation and can be influenced by fatigue and/or lack of collaboration. Therefore, electrophysiological methods (e.g. VEP, ERG, pupillometry) have been developed to study the visual field objectively [1, 2]. Previous studies have shown that measurements of visual evoked potentials (VEPs) elicited by multifocal stimuli can be used to detect visual defects [2–11].

VEPs can be subdivided in transient and steady-state responses based on the temporal content of the stimuli. In the transient onset-offset mode, signals are elicited by patterned stimuli that are presented at low temporal frequencies. Thus, the recordings consist of a response interval in which a stimulus is present and an interval in the absence of a stimulus and where the signal is determined by noise that is used for the estimation of a signal-to-noise ratio (SNR). Alternatively, a steady-state VEP is recorded when continuously stimulated with pattern reversals at a constant frequency of about 6 reversals/sec or higher. The amplitudes and phases of this steady-state response can be extracted from the Fourier spectrum at the reversal frequency. Noise, and thus SNR, can be obtained by using the amplitudes at non-stimulus frequencies [12, 13]. The steady-state VEP recording can be faster than the transient VEP because it is less susceptible to artefacts [14], and spectra can be evaluated more easily than amplitudes and delay times of troughs and peaks in transient VEPs [15, 16].

Multifocal VEPs (mfVEPs; i.e. VEPs elicited by multifocal stimuli) are mostly generated by sequential On–Off pattern presentations at several locations using a m-sequence technique. These mfVEPs are then of the transient type. As an alternative to stimulation with m-sequences, multifrequency stimuli have been suggested for recording steady-state mfVEPs (ssmfVEPs). When the recordings are performed with very high temporal resolution (i.e. using extremely high sampling rates), small bandwidths of response frequency components [13, 17] are possible. As a result, the responses to stimuli at different locations that differ only slightly in temporal frequency can be identified [4]. Ideally, the differences between stimulus frequencies are very small to ensure that the ssVEPs at the different locations originate from identical mechanisms. With different electrode settings, the recording with the best signal-to-noise-ratio (SNR) can be determined for further analysis. [17, 18].

It is the purpose of the present study to investigate whether multifrequency pattern reversal stimuli presented in different quadrants (QmfrVEPs) are suitable for detecting visual field defects in these quadrants. QmfrVEP measurements were performed in normal subjects and in glaucoma patients who showed visual field defects in quadrants. The results of the QmfrVEPs were correlated with perimetric data in the quadrants.

## Methods

### Stimuli

**Fig. 1:**
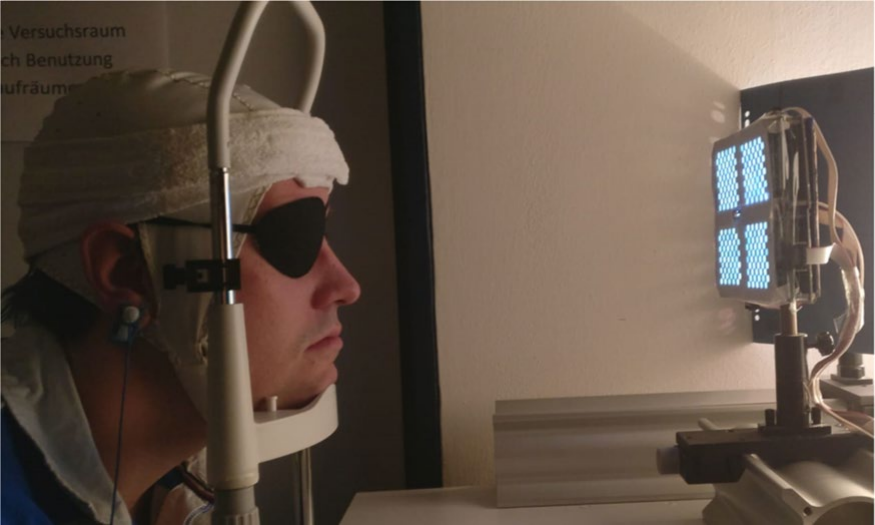
Experimental setup. The subject's monocularly focuses on a dim red LED, which is located in the middle of four arrays

**Fig. 2:**
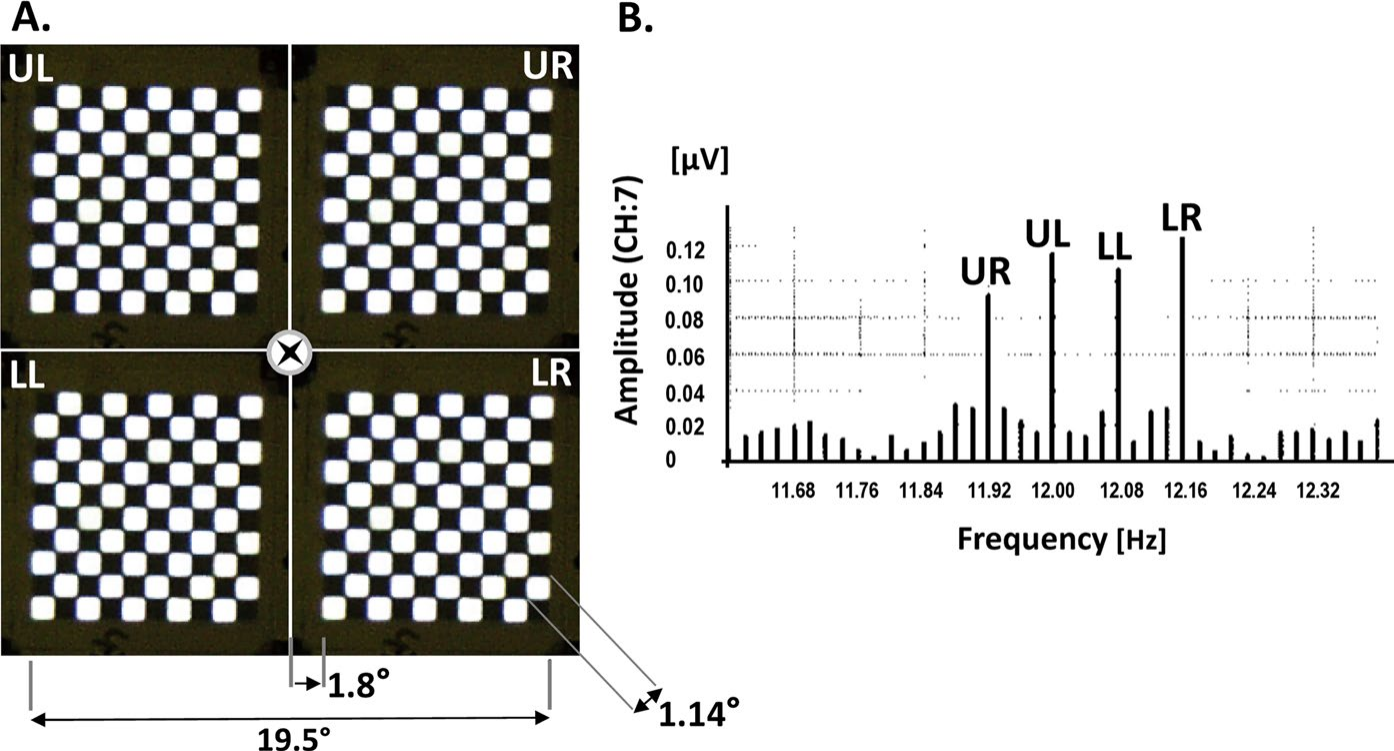
**A** Field of stimulation at a viewing distance of 32 cm. The side length of the arrays is 20.8°. The frame adds up to 1.9°. The arrays are denoted: Upper Left = UL, Upper Right = UR, Lower Left = LL, Lower Right = LR. **B** Amplitude spectrum of one channel (CH: 7) for a measurement with quadrant stimulation in a normal eye. The stimulus frequencies in the four arrays were: UL = 12.00 Hz, UR = 11.92 Hz, LL = 12.08 Hz, LR = 12.16 Hz. The amplitudes UR, UL, LL and LR correspond to the responses from the stimulus frequencies at the four arrays. The smaller amplitudes next to the stimulus amplitudes represent the random noise that was used for the SNR calculation

Pattern-reversal checkerboard stimuli were generated using four arrays of light-emitting diodes (LEDs; Roland Consult, Brandenburg, Germany). Figure 1 shows the measurement setup. A detailed description of the stimulus setup [19] and the diode arrays [20] was given previously. Briefly, the subject focused monocularly a fixation light in the middle of the four checkerboard arrays. The presently used viewing distance of 32 cm was based on pilot measurements in healthy subjects for obtaining optimal VEP recordings [21, 22]. The subject’s head was placed on a chinrest. Recordings were performed monocularly from the right/left eye randomly chosen. The four LED arrays were arranged so that they separately stimulated the four quadrants (Fig. 2A, Upper Left = UL, Upper Right = UR, Lower Left = LL, Lower Right = LR). Each array consisted of 100 LEDs in a 10 × 10 checkerboard layout with half of the diodes at maximal luminance and the other half switched off (Fig. 2A). The temporal modulation of the LED output was square wave. The size of a single LED was 4 × 4 mm. The diodes were separated by a 0.5 mm nontransparent material to prevent leakage of light, resulting in a contrast of almost 100%. At a viewing distance of 32 cm, the diagonal size of a single LED was 1.14° (spatial frequency: 0.88 cyc/deg). One LED array stimulated a retinal area of 8°. The margin between two neighbouring LED arrays was 3.6°. The space- and time-averaged luminance of an array was 170 cd/m^2^ as measured with a digital Photometer (Tektronix J16/J6503). The arrays were controlled by a FBGA generator (Reti-X, Kiessling Components, Chemnitz).

It has been shown before that a pattern reversal frequency between 10 and 12 reversals/sec elicited VEPs with maximal amplitudes [4]. Therefore, the stimulus frequencies in the four LED arrays were chosen to be close to 12.00 reversals/sec, but they differed slightly. At the nominal frequency of 12 reversals/sec the exact checkboard pattern reversal frequencies were 11.92 (UR), 12.00 (UL), 12.08 (LL) and 12.16 reversals/sec (LR). The time of one measurement was 50 s, resulting in 596 reversals at 11.92 reversals/sec, 600 reversals at 12 reversals/sec, 604 reversals at 12.08 reversals/sec and 608 reversals at 12.16 reversals/sec. To avoid onset artefacts, two single unrecorded pattern reversals preceded the recording in all measurements. Using spectral analysis, the responses to each quadrant stimulus were identified on the basis of its pattern reversal frequency, and the amplitudes and phases at the according frequencies were assigned to the corresponding quadrant (Fig. 2B). Clearly, the exact response frequencies corresponding to the pattern reversal frequencies in the stimulus (11.92, 12.00, 12.08 and 12.16 Hz) can be distinguished. To avoid fatigue, the room light was turned on for a minute between repeated measurements (4 or 5 repetitions). The measurements were performed with undilated pupils.

### QmfrVEP recording

**Fig. 3:**
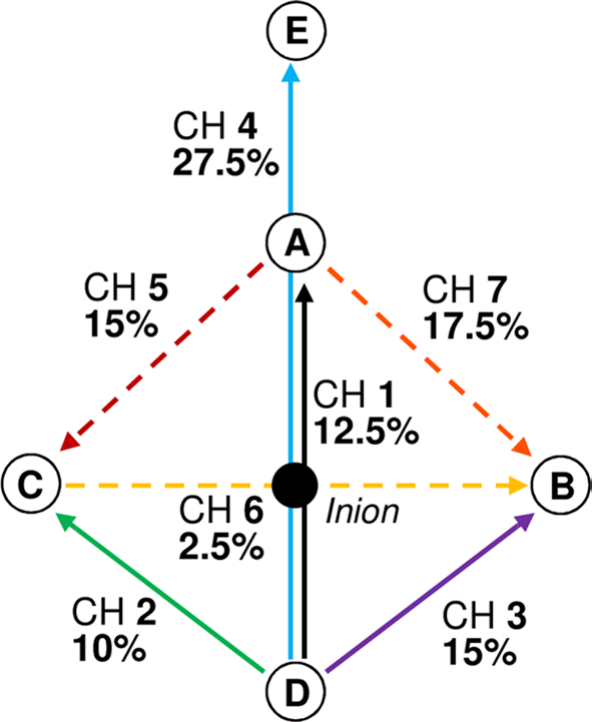
The present setup used five head electrodes and 7 channels. The electrodes A–C are located 4 cm right, left and above the Inion. Electrode D is fixed 2 cm beneath the Inion. An additional electrode E is placed 4 cm above electrode A. Dashed lines show virtual channels (5–7), calculated by real measured channels (1–3). The percentage of the records in which the concerning channel displayed the largest SNR is also shown

Pattern-reversal VEPs were recorded with a five electrodes setup using an electrode-fixating cap (Easycap GmbH, Herrsching am Ammersee, Germany). The reference electrode was 2 cm below inion (D), and the ground electrode was attached to the subject’s right earlobe. Similar to previous measurements [8, 12, 23, 24], three electrodes (A, B and C) were positioned at 4 cm distance above, left and right of the inion. An additional fifth electrode (E) was placed 8 cm above the inion [24] (Fig. 3). To reduce impedance, the scalp was cleaned with disinfectant (Sterilium, Paul Hartmann AG, Germany), and the upper callus was rubbed with Nuprep-scrub (Weaver, USA) before the cap was placed on the subject’s head. The electrodes were filled with electrode gel (GE medical systems Information + Technology, Germany).

Four channels (CH1: D–A; CH2: D–C; CH3: D–B; CH4: D–E) were recorded directly, while three additional virtual channels (Fig. 3: Dashed lines) were calculated offline by linear combinations from the direct recordings (CH5: A–C; CH6: C–B; CH7: A–B). The input signal was amplified 10,000-times and low-pass filtered with 70 Hz cutoff frequency (Gruber EMP88). To control the multifrequency stimulation and the VEP recordings, custom-written software (Reti-X, Kiessling Components, Chemnitz) was used. This system created the visual stimuli and allowed accurate analysis of the measured responses. Sampling rate was 1116 Hz. To increase the signal-to-noise ratio (SNR), the results of three or four recordings were averaged. The recordings were subjected to discrete Fourier transformation (DFT), and the amplitudes were obtained as a function of frequency. Owing to the recording time (50 s), the frequency resolution was 0.02 Hz, and for example, the 12 reversals/sec (6 Hz) stimulation used 186 samples for one cycle. For the SNR calculation, the amplitude of the signal at stimulus reversal frequency (*F*) was divided by the averaged amplitude at the neighbouring reversal frequencies to the left (NL1) and to the right (NR1) to the stimulus frequency (SNR1 = *F*/ (NR1 + NL1)/2). Hereby we assume that the stimulus does not elicit a response at these neighbouring frequencies, where the amplitudes are exclusively determined by noise.

From the seven channels, the recording with the maximal SNR (SNRmax) at the stimulus frequency was chosen for further analyses. Amplitude averages for noise calculation were scalar averages rather than vector averages also because SNRs were determined. Phases were neither considered in determining the diagnostic value of the method. In addition, the maximum amplitude of the seven channels was used for correlation analysis.

### Subjects and procedures

Measurements were performed monocularly in 5 glaucoma patients with visual field losses (5 females, range: 51–79 years old) and in 5 age-matched healthy subjects. Informed consent, including agreement for data collection, was obtained from all participants, after explanation of the purpose and the nature of the study. The study followed the tenets of the Declaration of Helsinki for research involving human subjects and was approved by the local ethics committee.

The patients were recruited from the “Erlangen Glaucoma registry’ (EGR) [25]. The patients of the EGR were clinically examined annually including applanation tonometry of the IOP (over the course of 24 h), morphometrical evaluation (Spectralis^®^ OCT) and functional tests (Octopus perimeter, program G1). There was no history of any other ocular disease, and no current or history of systemic diseases known to affect the eyes (e.g. diabetes mellites). To ensure an optimal accommodation, ametropia was corrected. Only recordings with complete outputs within the operating range of the amplifiers were analysed. Statistical analyses were performed using SPSS Statistics (SPSS-Inc., Chicago, USA) and the R language for statistical computing. The level of significance was 0.05. The correlation between SNR and perimetric visual defects of glaucoma patients was determined by the Spearman rank correlation. We used the R-package “effsize” for calculating Hedge’s g as an indicator for effect size independent of the underlying unit. To account for lack of independence between the four segments of each subject, we randomly drew 10^6^ samples from our 10 subjects (including the normal subjects) with replacement, and reported the median and the 95% confidence interval of the resulting Hedge’s g values (bootstrapping). Furthermore, we used the R-package “lmerTest” to fit a linear mixed-effects model for estimating the relationship between the QmfrVEP amplitude and the visual field while accounting for the interindividual variability and the small sample size. For this model, we used SNRmax as the dependent variable, perimetric quadrant defect as a fixed effect and patient as a random effect. The model did not allow for interaction between the fixed and the random effect, so that the same slope of the SNRmax versus perimetric defect plot was used for all patients.

## Results

**Fig. 4:**
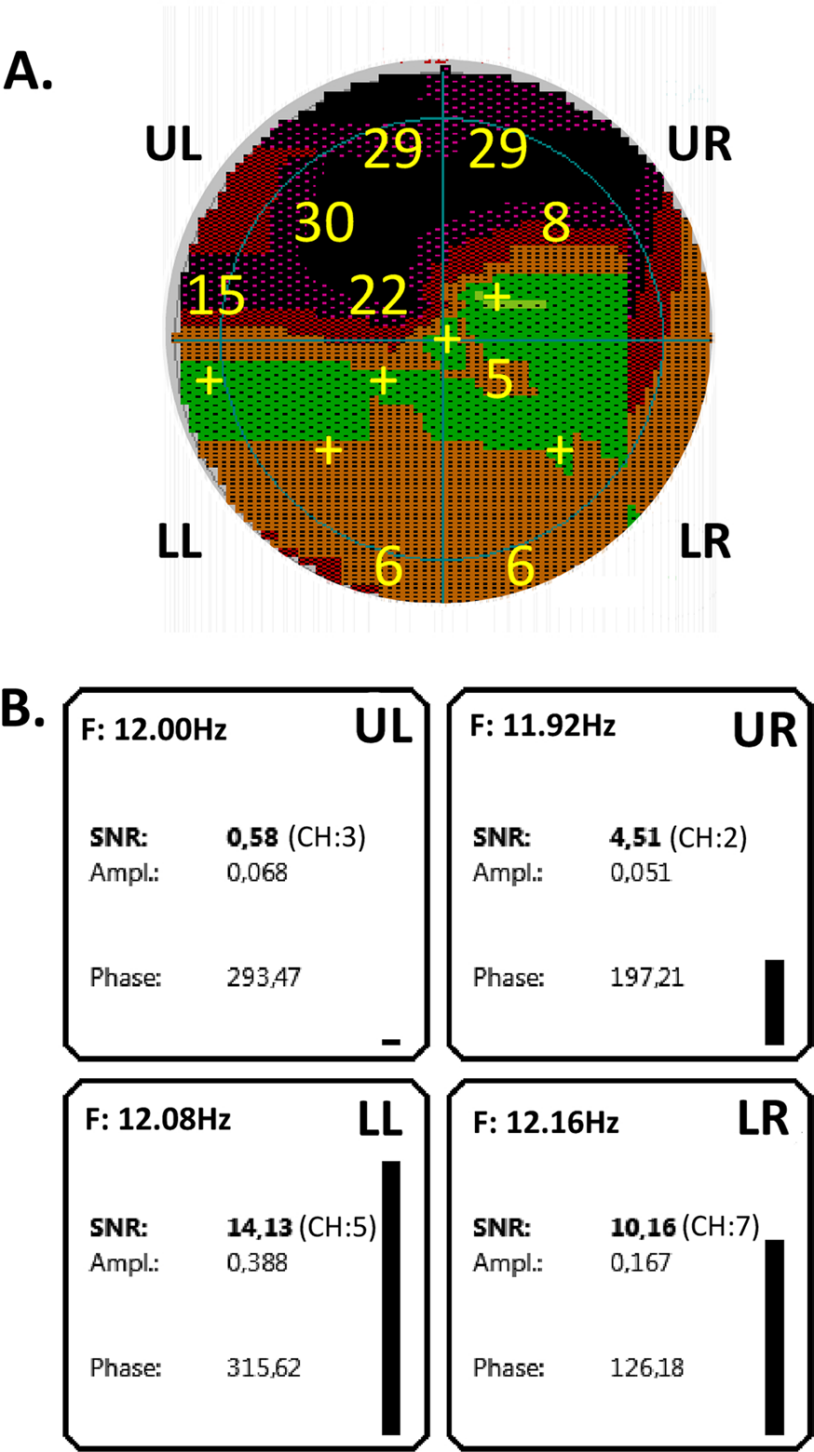
VEP and perimetry in a glaucoma patient. The figure shows screen prints of the present VEP software and Octopus G1. **A** Octopus perimetry of the central retinal area (12°) of the patient. Perimetry reveals advanced damages in the upper left quadrant. Here, defect depths of up to 30 dB are present. Also, in the upper right and lower right quadrants defects are observable, while the lower hemifield shows only minor perimetric defects. **B** "Results summary" of the present programme presents results for all LED arrays. For all quadrants, the "best-SNR" as well as the corresponding amplitude and phase is given. The channel from which the "best-SNR" has been derived is depicted in brackets. The columns at the side of each field indicate the level of the SNR graphically. In correspondence with decreasing perimetric losses (Fig. 4A), the SNR increases clockwise starting in the upper left quadrant (UL) until being the largest in the lower left quadrant (LL)

**Fig. 5:**
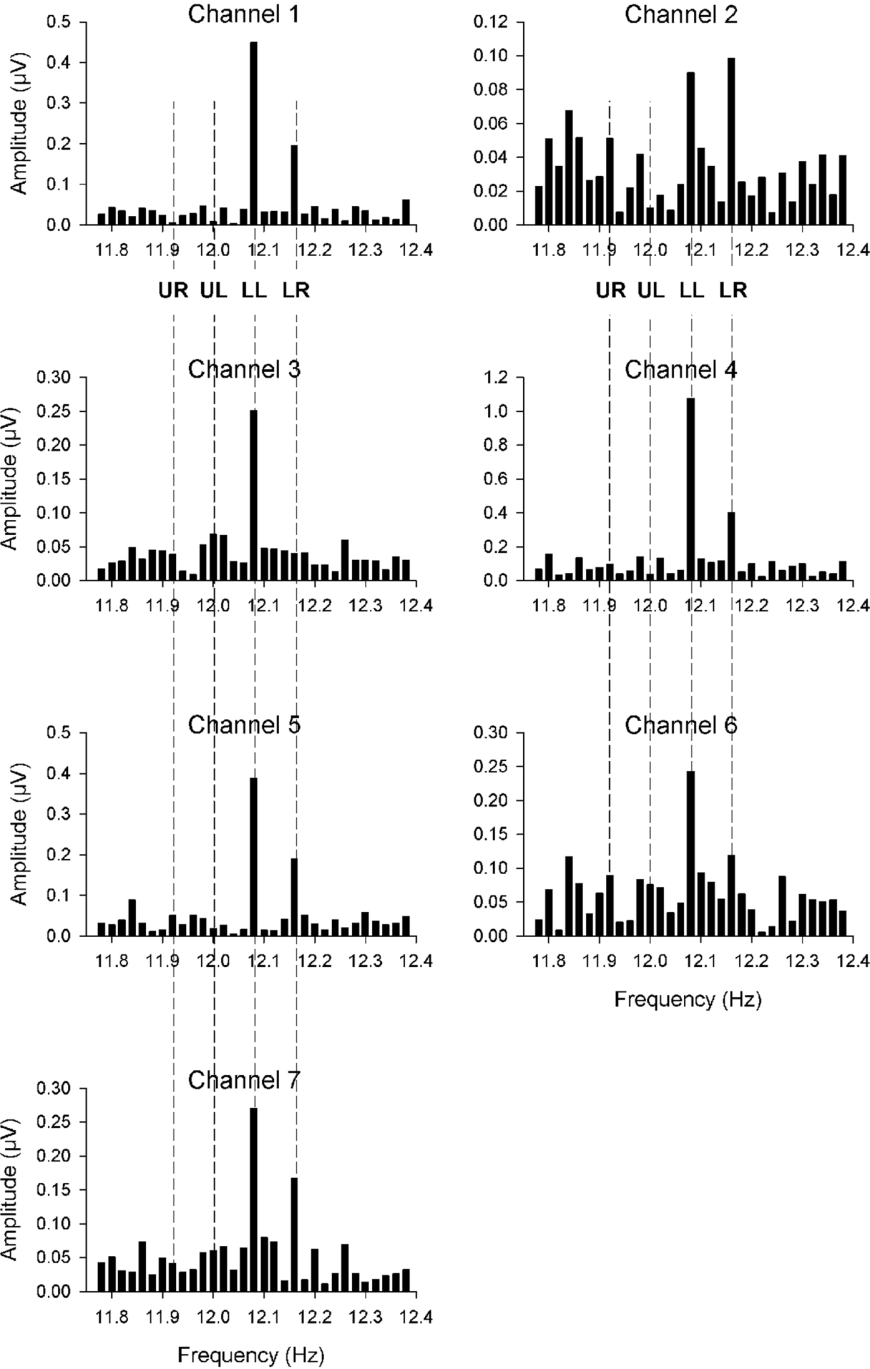
Spectra for 7 channels show high amplitudes for lower stimulus areas with normal visual fields. Amplitudes corresponding to the upper quadrants revealing perimetric defects are smaller

Figures 4 and 5 present results of a QmfrVEP measurement from a glaucoma patient with advanced perimetric losses. Octopus perimetry (Fig. 4A) in this patient resulted in averaged defects of 24 dB in the upper left quadrant, 18.5 dB in the upper right quadrant and in smaller defects in the lower quadrants. Figure 5 displays amplitude spectra of all channels. The response amplitudes from the lower left quadrant (*F* = 12.08 Hz) were large in all channels, while the response amplitudes from the upper left and upper right quadrant (*F* = 11.92 Hz, 12.00 Hz) were not above noise level in all channels. The responses from the lower right quadrant (at a frequency of 12.16 Hz) were intermediate. Please observe that the amplitudes were in qualitative agreement with the perimetric data.

In the subsequent analysis, the channel with maximal SNR was used for the quantification of a VEP defect (Fig. 4B). A visual field defect was associated with a lower SNR and vice versa. The upper left quadrant (stimulus frequency – 12 reversals/sec) with perimetric losses showed a very small SNR (0.58 dB). On the other hand, the lower left quadrant (stimulus frequency 12.08 reversals /sec) with nearly normal perimetry showed a VEP SNR of 14.13 dB. 

**Fig. 6:**
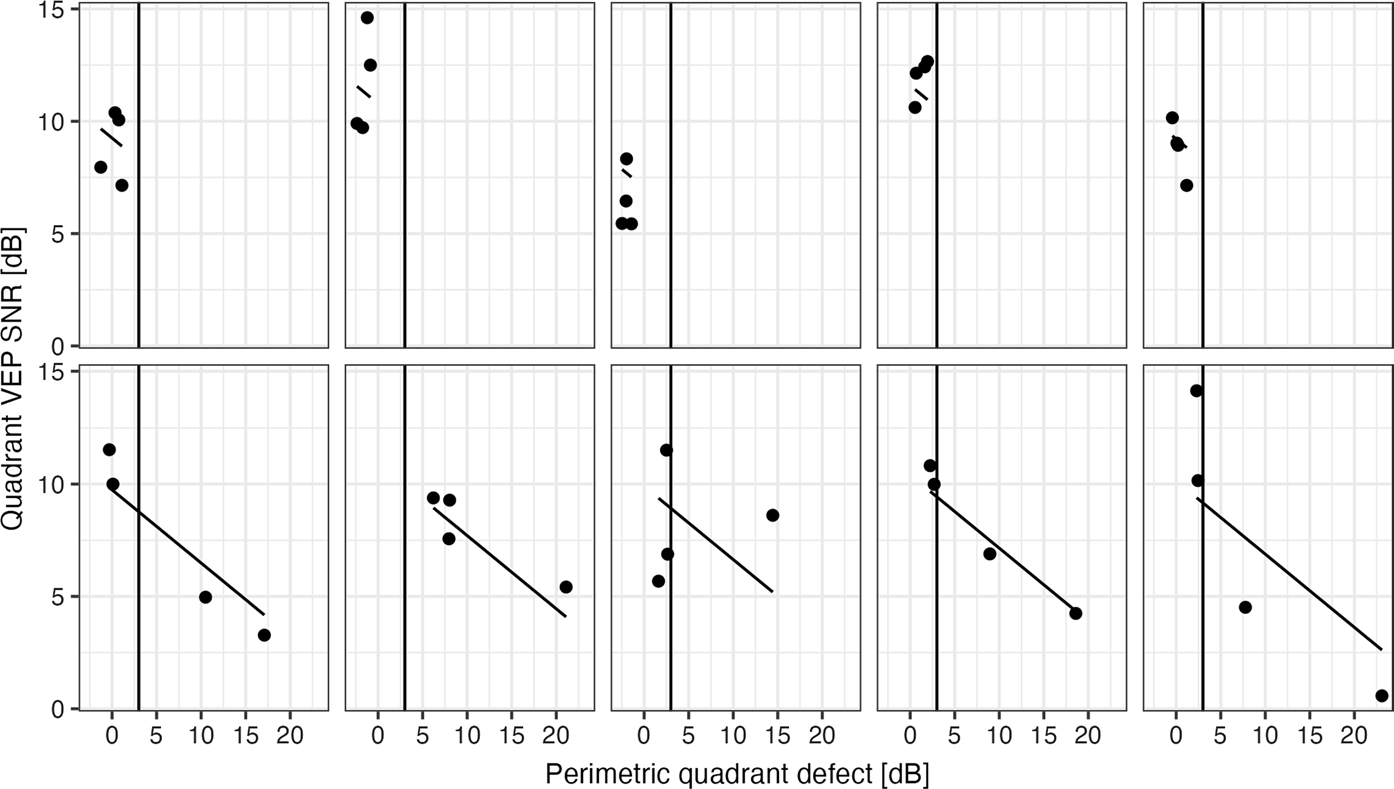
The scatterplots show the SNR results of the four quadrants individually for the five normal subjects (top row) and the five glaucoma patients (bottom row) as functions of the visual field defects. The vertical lines indicate the cutoff that separates “damaged” and “not damaged” quadrants. The other lines show the predictions from the linear mixed-effects model that was fit to the data. This model accounts for a fixed relationship between perimetry and VEP (the slope is equal for all patients), but also for a random overall variability in SNR between patients (variable intercept = patient effect). Note the considerable individual variability in the overall SNR. A clear relationship between perimetry and VEP can be observed in four patients, and the *p*-values were < 0.001 for the fixed effects

Figure 6 shows the relationship between SNRmax and the perimetric defects in each quadrant for the 5 healthy subjects (top row) and the 5 glaucoma patients (bottom row). The SNR is inversely correlated with the perimetric defect in the same quadrant. The effect of glaucomatous damage, defined as a quadrant perimetric defect > 3 dB, on the SNR yielded a large effect size (median Hedge’s g: 1.56, 95% confidence interval 0.51–3.10). This corresponds most likely to a large effect size, at least to a medium effect size. A spearman correlation test in the glaucoma patients test revealed that the two were strongly correlated (R =  − 0.76, *p* < 0.001). A similar analysis, using the maximal amplitudes (instead of SNRmax) from all channels (figure not shown), revealed a weaker correlation (*R* =  − 0.44, *p* < 0.05). Figure 6 also shows the predictions of the linear mixed-effects model, allowing a better appraisal of both the effect of glaucoma and the magnitude of inter- and intra-individual variability. The *p*-values for the fixed effects were < 0.001 for both the intercept and the slope, the standard deviation of the random effect (individual intercept) was 1.53. Altogether, the SNRs and the amplitudes were in agreement with the perimetric data.

## Discussion

### Clinical implications

In the present study, SNRs and (to a lesser degree) amplitudes obtained with QmfrVEPs were correlated with visual field losses in glaucoma patients. This shows that the SNR can be used as a direct outcome parameter to quantify functional losses and not only as an indicator for signal quality.

All segments with SNR values above 10 dB had normal sensitivity in the corresponding visual field locations (“undamaged” segments). However, some normal subjects had SNR values of similar magnitude as in segments that included locations with visual field defects in the glaucoma patients (“damaged” segments). Therefore, QmfrVEP may allow identification of local defects in glaucoma patients and objective demonstration of normal function when SNR values are high, but the prediction of absolute sensitivities in the visual fields may be limited by the large interindividual variability of the VEP.

It has been shown before that reduced mfVEP amplitudes can be an early predictor of axonal damages [11] and can be used to detect visual field losses in unilateral optic neuritis [8], temporal hemianopia [8, 21] and glaucoma [26]. In addition, patients who display structural damage in OCT images showed changes in mfVEP. Furthermore, the magnitude of mfVEP defects was correlated with retinal nerve fibre layer thickness [21]. In addition, mfVEP has previously been found to give more comprehensive information about damage in small parts of the visual field, than the VEP [27].

The small sample size of five patients limits the applicability of parameters like sensitivity and specificity. However, our data show a large effect size of SNR when comparing damaged and undamaged segments.

Currently, mfVEP recordings are not used in clinical routine due to the considerable interindividual variability of the mfVEP amplitudes [2, 7, 26, 28, 29] and due to the absence of a “gold standard” [8]. Possibly, the multifrequency technique, as presented in the current study, is a useful alternative to obtain objective field losses because the evaluation of the signals is easier and less prone to errors than measuring single peaks in transient responses. Further studies including larger cohorts of subjects are required to assess sensitivity and specificity for detecting subjects with visual field defects.

The QmfrVEP can possibly be used in other diseases. It would be particularly interesting to study whether the SNR improves in parallel with the visual field in diseases with reversible defects (e.g. compressive neuropathy due to a pituitary tumour measured pre- and postoperatively). Repeated measurements in progressive visual field losses might uncover the diagnostic utility of amplitudes and SNRs in follow-up of these patients.

The response phases can also be obtained from the DFTs but were not analysed in the current study because only phase values measured at the same channel can be compared. Phases strongly vary between different individuals and between different stimulus locations. However, the phases may be an interesting additional parameter in single patients to monitor disease progression or the effects of therapeutic intervention.

### Optimizing stimulus parameters

Our measurements show that QmfrVEPs can provide information from several retinal locations simultaneously. Improvement of this technique by an increase of the number of stimulated retinal locations may be a further development to reach an objective perimetry. Earlier, Abdullah and coworkers [22] were able to demonstrate the possible usefulness of 9 and (to a minor degree) 17 stimulation fields.

In the present study, four visual quadrants were stimulated using checkerboard devices of fixed geometry. Thus, different field sizes of the stimulated retinal areas could only be achieved by changing the distance between stimulus and observer or with stimulators showing other geometric data. If peripheral locations are to be included, the stimuli should correct for eccentricity dependent cortically scaling [30].

In addition, if more stimulus areas are needed the frequency differences in the stimuli should be small enough to ensure that all VEPs originate in the same cortical mechanism. If a high number of stimulus areas is used, the recording time should be increased accordingly in order to able to achieve sufficient temporal resolution and to distinguish the responses and SNRs elicited by the different arrays. However, it should be noted that, in contrast to the m-sequence-based mfVEP, interruption and restart of measurements are not possible, because a complete measurement is necessary to perform the DFT. Therefore, recordings with large artefacts (especially where the signals were outside of the amplifier’s limits) must be considered carefully. An improvement of the SNR can be achieved by averaging repeated traces before spectrum analysis.

In addition, all stimulus frequencies should be sufficiently different from those at which alpha waves occur (i.e. at about 10 Hz) [12] and should be chosen outside this frequency range. Preceding studies showed that a stimulus frequency of about 12 Hz, as used in the present student, gives optimal results [19, 28, 31–33].

As in an earlier study [19], we used the ratio of signal amplitude at reversal frequency divided by the mean of two neighbouring frequencies for calculations of the SNR ratio [14]. Pilot studies showed that the use of two direct neighbouring frequencies for SNR calculation performed better than the inclusion of 4 or more [34] non-signal amplitudes.

One disadvantage of the method is on the one hand the long duration (50 s per run) and on the other hand the high number of repeats. If we succeed in using dichoptic stimuli, it will allow us to reduce the test duration. A disadvantage of the objective technology in comparison to standard perimetry is the time-consuming preparations for placing the electrodes. Compared to the usually applied electrode configuration in multifocal VEP measurements, we used one additional central electrode. This additional electrode was most frequently (27.5%; see Fig. 3) chosen as best channel from the best-of-algorithm. However, if this additional electrode was excluded from correlation between SNR and perimetric losses only a small reduction of the correlation coefficient was seen (seven channels: − 0.76, six channels: − 0.73).

Finally, it should be taken in consideration that our LED arrays used light tight material between the LEDs achieving a contrast of nearly 100% [19]. This could mask visual defects of patients with glaucoma disease, because of saturating damaged and undamaged quadrants simultaneous. Therefore, future measurements with reduced stimulation contrasts [22] might be helpful to improve discrimination.

## Conclusion

QmfrVEPs can possibly be applied in ophthalmology, neurology and neurosurgery to analyse visual field damage objectively and to evaluate the success of visual pathway surgeries (e.g. pituitary surgery).

## Data Availability

The results presented in the manuscript are part of the dissertation by Katja Crux.
